# Encoding and Recognition Processing of Chinese Characters: A Functional Magnetic Resonance Imaging Study

**DOI:** 10.1155/2016/5983671

**Published:** 2016-01-11

**Authors:** Jinlong Zheng, Siyun Shu, Bin Wang, Xiangyang Tian, Xinmin Bao, Yongming Wu, Zengqiang Zhang, Xiangyang Cao, Lin Ma

**Affiliations:** ^1^Department of Neurology, Huai'an First People's Hospital, Nanjing Medical University, Huai'an, Jiangsu 223300, China; ^2^Center of Pediatrics, Zhujiang Hospital, Southern Medical University, Guangzhou, Guangdong 510282, China; ^3^Department of Neurology, Nanfang Hospital, Southern Medical University, Guangzhou, Guangdong 510515, China; ^4^Institute of Geriatrics and Gerontology, Hainan Branch of Chinese PLA General Hospital, Sanya, Hainan 572014, China; ^5^Department of Radiology, PLA General Hospital, Beijing 100853, China

## Abstract

This study aimed to investigate the conceptual memory processes that underlie encoding and recognition processing of Chinese characters. Healthy participants (*n* = 14) performed a semantic-relatedness paradigm using categorically related logogram pairs from four different categories (fruit, animal, tool, and clothing). During intentional encoding, subjects were instructed to make semantic judgments and select category-correlated features to bind and memorize logogram pairs. During recognition, subjects were asked to recognize the memorized items. The MATLAB software and spatial clustering analysis were used for image data processing. Compared with baseline, encoding mainly activated BA13, with significant effects in BA6/8/9/46/45/47, BA24, BA7/39/40, BA37/20, and BA18/19; meanwhile, recognition mainly activated BA6/8/9/10/13/45/46/47, BA31, BA7/40, and BA18/19. Compared with recognition, encoding activated BA18/19/37/20/36 with a peak activation area in BA18. Compared with encoding, recognition significantly activated BA7, BA31/32, and BA10. In conclusion, distributed networks of discrete cortical regions with distinct roles are active during semantic processing of logograms. The ventral occipitotemporal and inferior frontal regions display increased levels of encoding-related activity. The dorsal medial brain regions, including the superior frontal gyrus and occipitoparietal regions, are associated with recognition-related activity.

## 1. Introduction

Conceptual memory is involved in the general knowledge of concepts, objects, facts, people, and word meaning. Recent neuroimaging studies in conceptual knowledge begin to reveal that specific functions are performed within small areas of the brain, with these small areas involved in a hub or network that also includes areas of the brain responsible for perceiving and acting [1]. Conceptual knowledge is usually shared by individuals from the same culture, with some variations according to each individual experience.

Alphabetic languages and logographic Chinese are markedly different because they represent the meaning of words in very different ways. Indeed, alphabetic words are read by assembled phonology, while Chinese characters are read as a concept by their visual shapes, without involvement of phonemes [2]. A previous study revealed that phonological processing of Chinese characters occurs in Brodmann area (BA) 9, BA46, and BA40, with minor activation of BA45/47, while phonological processing in native English speakers is made in BA44/45 and BA22 [3].

However, the organization of this knowledge within the brain is controversial. Indeed, there are two theories about this: the distributed-only and distributed-plus-hub views, respectively [1]. Studies revealed that recognizing specific knowledge activates specific areas: colors and forms activate the left ventral temporal cortex, whereas recognition of size information activates the parietal cortex [4–6]. Tasks such as animal naming are uniquely associated with the left medial occipital cortex, whereas tool naming specifically involves the left premotor and posterior middle temporal cortices [7, 8].

Recent studies have provided strong evidence that the human conceptual system is not organized by category specificity [9, 10]. It has been postulated that the organization of semantic representations has more than one governing principle. It is important to note that the activity associated with each object-specific category involves a relatively large expanse of brain areas, suggesting that representations of different categories are distributed and overlapping [1]. It is possible that feature-based models provide the flexibility needed to represent an infinite variety of object categories [11, 12].

Few studies have examined the brain areas involved in the processes of encoding and recognizing conceptual information regarding Chinese characters. Therefore, the aim of the present study was to use functional magnetic resonance imaging (fMRI) to study the brain areas involved in encoding and recognizing conceptual information concerning Chinese characters.

## 2. Materials and Methods

### 2.1. Subjects

Fourteen native Mandarin Chinese speakers, including seven males and seven females, were recruited to participate in the semantic category memory test. All subjects were right-handed as determined by the handedness inventory [13]. Participants were all undergraduate students from the Southern Medical University, Guangzhou, China. Participants ranged in age from 20 to 23 years and had normal or corrected-to-normal vision. This study was approved by the Medical Ethics Committee of Nanfang Hospital. All participants provided written informed consent before enrolment, in accordance with the Declaration of Helsinki. The subjects underwent a Clinical Dementia Rating (CDR) test. Inclusion criteria were (1) no memory disturbances, (2) normal physical status, and (3) CDR = 0. Subjects who had a history of neurological or psychiatric illness condition or who were taking any medications immediately prior to or during the scans were excluded from the study.

### 2.2. Chinese Characters

All test logograms (visually presented in pairs to each subject) were selected from* the Modern Chinese Dictionary* [14]. All logograms were used commonly and had a frequency of occurrence of no less than 30 per million, according to* the Modern Dictionary of Frequently Used Chinese* [15]. In each logogram pair, the first one was regarded as the stimulating logogram, while the second was considered the responding logogram. Four groups of semantic categories (fruits, animals, tools, and cloths) were selected for this study, using four stimulating and four responding logograms from the same category, alongside eight other logograms from similar categories. By combinations and repetitions, a total of sixty-four logograms were selected for each category memory task.

### 2.3. fMRI

The experiment was performed using a 1.5 Tesla Magneto scanner (Siemens, Sonata, Germany) equipped with a fast gradient system for echo-planar imaging (EPI). A standard radio frequency head coil was used. Head motion was restricted, with ear plugs used to reduce scanner noise. Visual stimuli were presented to subjects by projecting the video display of computer onto a translucent screen. Subjects viewed the stimuli through a mirror attached to the head coil. The presentation time of stimuli was accurately controlled by the DMDX display software (http://www.u.arizona.edu/~kforster/dmdx/dmdx.htm). Before the tests, subjects were visually familiarized with the procedures and test conditions to minimize anxiety and to enhance task performance. EPI was performed using a gradient-echo with scan parameters of repetition time (TR)/echo time (TE)/flip angle = 2,500 ms/25 ms/90 degrees. The acquisition of the matrix was 64 × 64 in each plane, with a field-of-view (FOV) of 210 mm × 210 mm. Thirty contiguous axial slices (slice thickness = 4 mm) parallel to the AC-PC line were acquired to cover the whole brain. Anatomical MRI was acquired using a T1-weighted 3D gradient-echo pulse-sequence, which provided high resolution (1 × 1 × 1 mm^3^) images of the entire brain.

### 2.4. Presentation of Logogram Pairs

**Figure 1:**
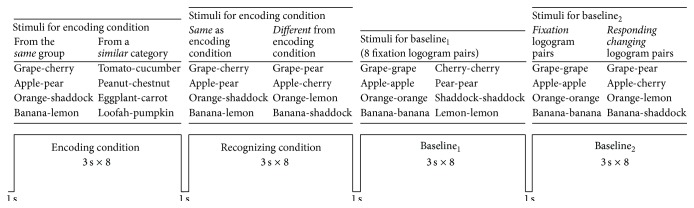
Design of one stimulation cycle. Each cycle contained four blocks. Each block was preceded by a 1 s period during which an image was presented to remind the patient to the study principle. Then, eight pairs of logograms were displayed for 3 s each. The figure used the fruit category as an example. The logogram pairs appeared in a random order.

All logograms were presented to the subjects in Chinese using the same font and size. A block design was used. The test in each subject contained four cycles composed of four blocks: 25-second (s) block of a memory-encoding condition, 25 s block of a recognition condition, 25 s block of a baseline_1_ condition, and 25 s block of baseline_2_ condition. Each block consisted of an image reminding the patients of the testing process displayed for 1 s, followed by eight logogram pairs displayed for 3 s each (Figure 1). Each cycle covered one category: fruits, animals, tools, or cloths.

During each memory-encoding condition block, subjects were asked to identify and memorize four logogram pairs that belonged to the same category (e.g., fruit group test:* grape-cherry*), and these were mixed with other four similar logogram pairs (e.g.,* tomato-cucumber*). During intentional encoding, subjects were instructed to make semantic judgments and select category-correlated features to bind and memorize logogram pairs; that is, subjects were instructed to judge the same group of logogram pairs from the presented eight pairs using their own conceptual knowledge (e.g., size, color, and form) to find the stimulating logogram and responding logogram and memorize them. Each logogram pair was presented randomly for 3 s.

In each recognition condition block, all pairs of logograms shared the same category (e.g., fruit group). There were four logogram pairs presented that were exactly the same as during the encoding condition; the other four logogram pairs were recombined from the same group (e.g.,* grape-cherry and grape-pear*). Examples of stimuli for the fruit group test are presented in Figure 1. The logogram pairs appeared in a random order. Subjects were required to recognize four pairs of logograms just memorized during the encoding condition. Subjects indicated their logogram pair selection by pressing a key button with the index of the right (dominant) hand.

In each baseline_1_ condition block, a fixation logogram pair (e.g.,* apple-apple*) was adopted in order to create a condition in which no memorization or recognition was needed. The fixation logogram pair was replaced every 3 s, and subjects were instructed to maintain fixation on the logogram pair.

In each baseline_2_ condition block, four fixation logogram pairs (e.g.,* apple-apple*) were presented together with four other logogram pairs, with the responding logogram different from the stimulating one (e.g.,* apple-cherry*). The button had to be pressed when the fixation pair appeared.

### 2.5. Data Analysis

The MATLAB software (MathWorks, Inc., Natick, MA, USA) and SPM8 (http://www.fil.ion.ucl.ac.uk/spm) were used for image data processing. Image preprocessing was performed using the Data Processing Assistant for Resting-State fMRI (DPARSF) V1.0 software (http://restfmri.net/forum/DPARSF). DPARSF is a plug-in software package based on SPM (http://www.fil.ion.ucl.ac.uk/spm) and RS-fMRI Data Analysis Toolkit (REST, by Song et al., http://www.restfmri.net) [16]. Functional data preprocessing included slice timing correction, motion correction, spatial normalization, and smoothing (FWHM = 4 mm). Data with head motions of more than 2.0 mm maximum displacement in any of the *x*, *y*, or *z* directions or 2.5 degrees in any angular motion were discarded.

Preprocessing of fMRI data was performed with a time-series of images acquired from the same subject using the least squares approach and six-parameter (rigid body) transformation to remove movement artifacts. The images were spatially smoothed with an 8 mm Gaussian kernel to decrease spatial noise. Skull stripping of the 3D MRI T1-weighted images was carried out with the Alice software (Perceptive Systems, Inc., Boulder, CO, USA). A generalized linear model approach was used in each participant according to image groups (encoding, recognition, baseline_1_, and baseline_2_). Activation maps were calculated by comparing images acquired during the task with control conditions (i.e., the encoding condition was compared with baseline_1_ and the recognition condition with baseline_2_) using Student's *t*-tests. One-sample *t*-tests were performed on activation maps using SPM8 (http://www.fil.ion.ucl.ac.uk/spm). Multiple comparison corrections for the data were performed using the Monte Carlo simulation (AlphaSim by B. Douglas Ward, http://afni.nimh.nih.gov/pub/dist/doc/manual/AlphaSim.pdf). Combination threshold of voxel was *P* < 0.05, and a cluster size >389 corresponded to a corrected *P* < 0.05.

The activation maps of all 14 subjects were then overlaid on the corresponding T1 images using random effect analysis [17].

MNI coordinates were converted to Talairach coordinates using nonlinear transformation. Coordinates shown in Talairach space for the center-of-mass and volume (mm^3^) of each activation cluster were determined based on the averaged activation maps. Anatomical labels and BAs were identified according to the Talairach Daemon database. The regions that showed significant differences were extracted as regions of interest (ROIs).

## 3. Results

**Figure 2:**
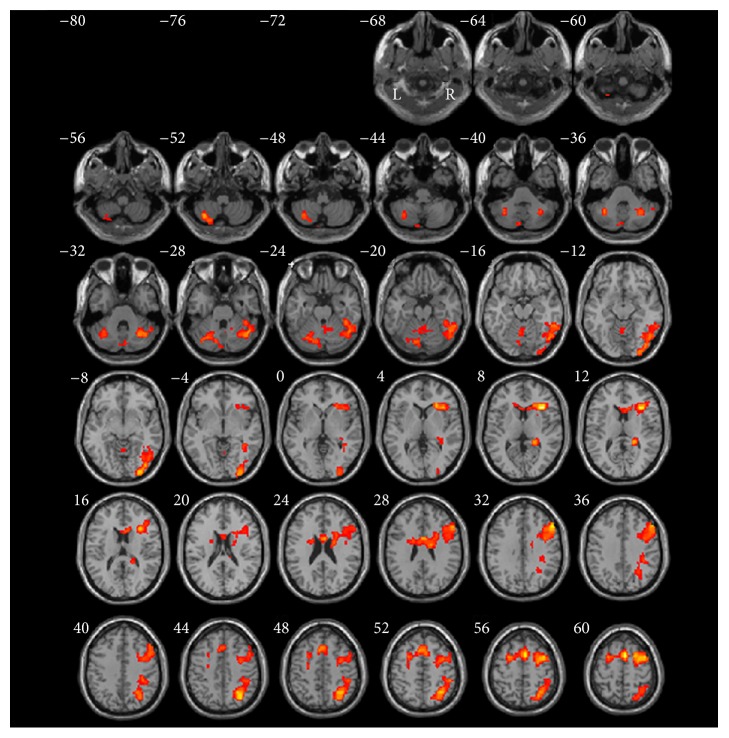
Normalized brain activation maps averaged over 14 participants at a threshold of *P* < 0.05 of encoding versus baseline_1_. Talairach *Z* coordinates are given above each horizontal section. Combination threshold of voxel was set at *P* < 0.05, and a cluster size >389 corresponded to a corrected *P* < 0.05. This correction was determined using the AlphaSim software in AFNI. L, left hemisphere; R, right hemisphere.

The detailed brain regions of significant activation for encoding versus baseline_1_ are listed in Table 1 and presented in Figure 2. When comparing encoding with baseline_1_, large clusters of activation were located in right prefrontal cortex with a peak activation area in insular cortex (BA13) extending to the midinferior frontal region (coordinates: 36, 24, 7, *t*-value = 9.20, and *P* < 0.05). Significant activations included three clusters in different regions: (1) right prefrontal cortices (BA6/8/9/46/45/47) including sublobar/claustrum and anterior cingulate gyrus (BA24), their activation extending to the prefrontal area; (2) right parietal lobe (BA7/39/40); and (3) ventral occipitotemporal cortex including fusiform gyrus (BA37/20) and inferior occipital cortex (BA18/19).

**Figure 3:**
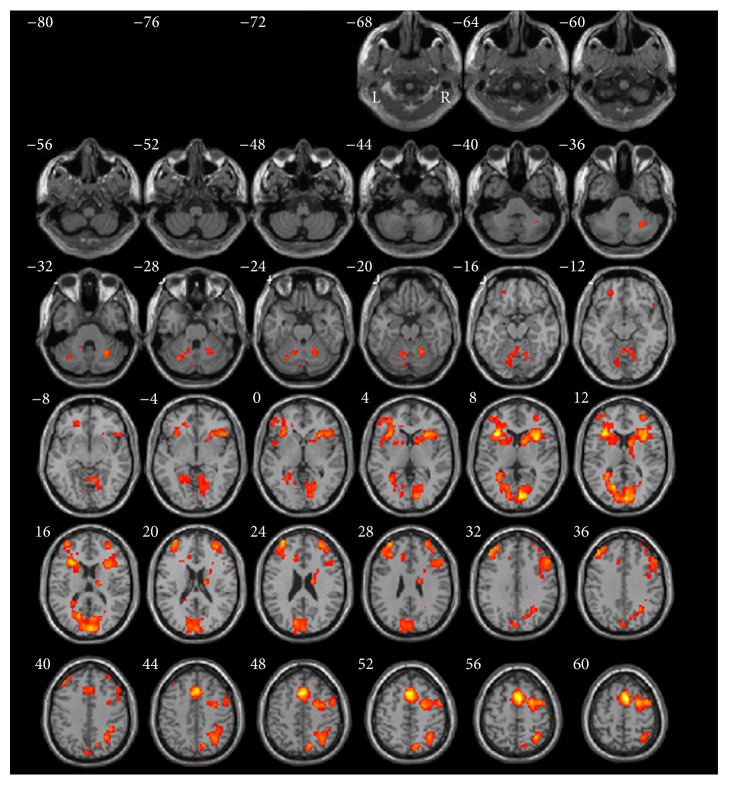
Normalized brain activation maps averaged over 14 participants at a threshold of *P* < 0.05 of recognition versus baseline_2_. Talairach *Z* coordinates are given above each horizontal section. Combination threshold of voxel was set at *P* < 0.05, and a cluster size >389 corresponded to a corrected *P* < 0.05. This correction was determined using the AlphaSim software in AFNI. L, left hemisphere; R, right hemisphere.

As shown in Table 1 and Figure 3, when comparing recognition versus baseline_2_, the activation pattern in bilateral cortices included frontal cortex (BA6/8/9/10/13/45/46/47), posterior cingulate gyrus (BA31), parietal lobe (BA7/40), and cuneus (BA18/19). However, there were limited activities in left cortices. Significant activations included two clusters in different regions: (1) bilateral superior medial frontal and (2) occipitoparietal regions. Strong activation was located in the right superior frontal gyrus with a peak activation area in the premotor cortex (BA6, coordinates: 6, 9, 58, *t*-value = 8.54, and *P* < 0.05).

**Figure 4:**
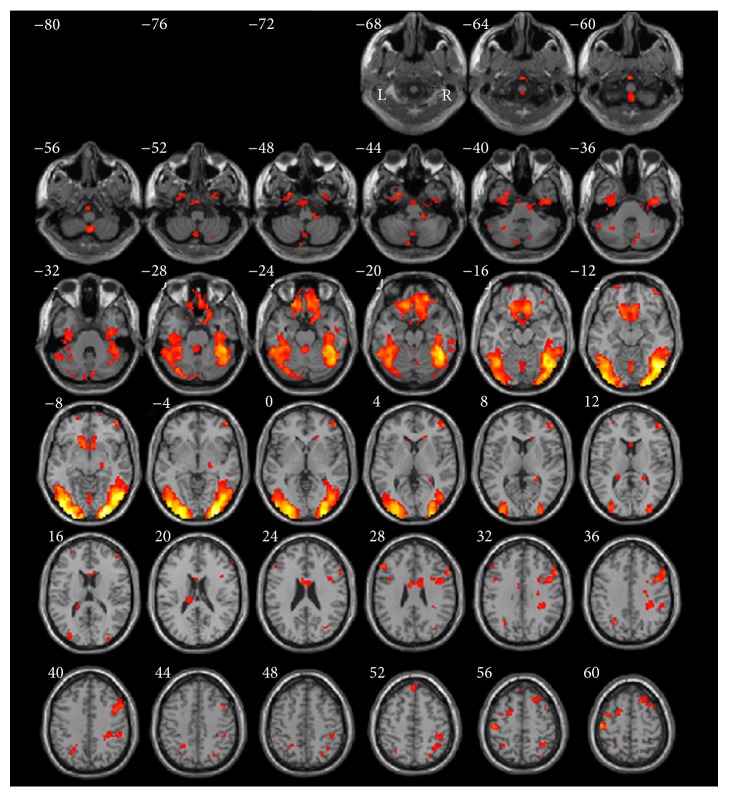
Normalized brain activation maps averaged over 14 participants at a threshold of *P* < 0.05 of encoding versus recognition. Talairach *Z* coordinates are given above each horizontal section. Combination threshold of voxel was set at *P* < 0.05, and a cluster size >389 corresponded to a corrected *P* < 0.05. This correction was determined using the AlphaSim software in AFNI. L, left hemisphere; R, right hemisphere.

The detailed brain regions of significant activation for encoding versus recognition are presented in Table 2 and Figure 4. Compared with recognition, encoding activated large clusters in bilateral ventral occipitotemporal cortex (BA18/19/37/20/36) with a peak activation area in left inferior occipital gyrus (BA18) (coordinates: −30, −96, −3, *t*-value = 9.22, and *P* < 0.05). Significant activations included two clusters in different regions: (1) ventral occipitotemporal cortex and (2) inferior and medial frontal cortices (BA47/11/25).

**Figure 5:**
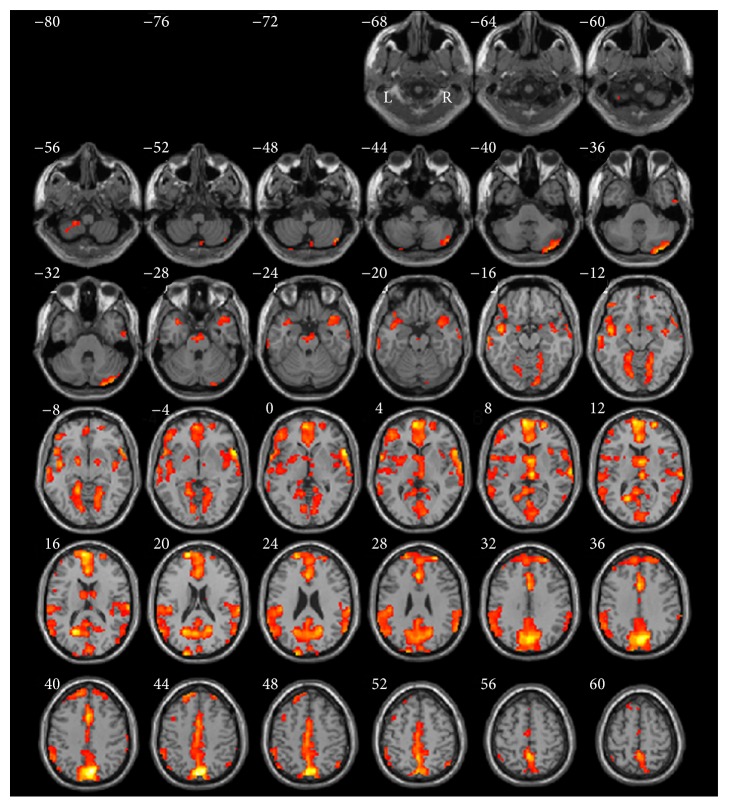
Normalized brain activation maps averaged over 14 participants at a threshold of *P* < 0.05 of recognition versus encoding. Talairach *Z* coordinates are given above each horizontal section. Combination threshold of voxel was set at *P* < 0.05, and a cluster size >389 corresponded to a corrected *P* < 0.05. This correction was determined using the AlphaSim software in AFNI. L, left hemisphere; R, right hemisphere.

As shown in Table 2 and Figure 5, compared with encoding, recognition significantly activated three clusters in different regions: (1) bilateral precuneus (BA7), (2) cingulate gyrus (BA31/32), and (3) superior medial frontal gyrus (BA10). Strong activation was located in the left superior parietal gyrus with a peak activation area in precuneus (BA7, coordinates: −3, −73, 45, *t*-value  =  8.50, and *P* < 0.05).

## 4. Discussion

The present fMRI study aimed to explore neural substrates of integrative semantic categories in a more holistic manner. In contrast to previous studies, we used category-related logogram pairs to contribute to deep semantic strategies for encoding and recognition tasks. To ensure that brain activation was specifically attributed to semantic processing and not to categorical processing, semantics and control conditions were matched for orthographic or phonological (in Chinese) processing demands, and all four categories were pooled together. Logogram pairs from four different categories (fruits, animals, tools, and cloths) were included in a single experiment and analyzed as an entity to examine cerebral activity at a relatively integrative level rather than at a specific level.

The tasks employed in the present study activated ventral occipitotemporal, inferior frontal, superior frontal gyrus, and occipitoparietal regions, but not the posterior left inferior frontal gyrus (LIFG) (BA44/45), which is commonly activated in semantic memory tasks. The role of the LIFG remains unclear. Some studies claimed that the anterior LIFG (BA47) plays an important role in semantic processing [18], whereas the posterior LIFG (BA44/45) is specialized for phonological processing [19]. The data presented above suggest that the anterior region is associated with semantic processing, irrespective of phonological demands. The LIFG has been previously shown to be involved in generating semantic associations [20, 21], particularly while making decisions concerning semantic associations [22–24]. Another explanation of this modulatory response is that it reflects increased demand for selection between categorical associations [25–27]. In the semantic tasks used in the present study, the subjects were required to select the related features of the logogram pairs to generate categorical associations. Therefore, our findings support the idea that the LIFG is involved in selecting among competing semantic features stored in the cortex [28].

The dorsolateral prefrontal cortex (DLPFC) is roughly equivalent to BA9 and BA46 and plays an important role in processing mnemonic information, for example, working memory. Some fMRI data have shown that the DLPFC activity is higher during semantic relationship-encoding processing compared with item-specific encoding [29–31], indicating that the DLPFC preferentially modulates semantic relational processing. In this study, subjects were required to build semantic relations between items using category-correlated features. Thus, it may be inferred that left BA9/46 activation may contribute to modulating semantic relational processing, as might be suggested by previous studies [32, 33]. Nevertheless, these results are similar to a previous study showing that BA9/46 is involved in the recognition of Japanese logograms [34].

As shown above, the total extent of brain activation, particularly in lateral-ventral regions of the occipitotemporal cortex, was significantly larger for the encoding process compared with the recognition one. These results indicate that the neurocognitive mechanisms of semantic processing underlying encoding may differ from those behind recognition [35]. Goodale and Milner [36] proposed a division of labor in the visual pathways of primate cerebral cortex between a ventral stream that contributes to perception of the visual world and a dorsal stream specialized for the visual control of action. In the current study, lateral and ventral occipitotemporal regions displayed increased levels of encoding-related activity when the subjects were required to encode logogram pairs. However, we found little evidence for ATL involvement in semantic processing; activation was only observed in the ventral-lateral regions of posterior temporal cortex. This is consistent with a report showing that normal individuals display strong activation of ventral-lateral regions of posterior temporal cortex during a categorical association task, whereas patients with ATL atrophy failed to show activity in this region [37].

Our data suggest that the encoding task relies on feature-general processes to identify target word pairs for memorization and feature-specific processes to encode the pairing, while the retrieval task only relies on feature-specific processes for successfully retrieving the response words. The direct contrast between these two tasks might reflect the feature-general processes of semantic representation. However, further study using Chinese characters is necessary to complement these results. A previous report proposed that adults more effectively engage right hemisphere brain regions involved in the visual-spatial analysis of Chinese characters [38], indicating that Chinese character processing is age related. In addition, semantic and visual errors were shown to be associated with young age and low education level in children [39]. Therefore, in this study, we selected subjects with similar ages and education levels, with the examination process clearly explained to all subjects, to minimize the effects of these confounding factors. Further studies will focus on populations of various ages as well as different language comprehension and education levels.

The present study suffers from some limitations. It was not exhaustive in semantic scope since all four categories were pooled to only keep the effect of encoding and recognizing logograms. It would be interesting to test subjects with the same method on different tasks, such as categorically related picture pairs. It would also be useful to examine the anatomic and functional connectivity of the ROIs to identify the directional and effective connections between ROIs, thus determining how ROIs interact with each other within a broader network. Such connectivity analysis can be explored to assess whether anatomic connectivity affects functional connectivity. Finally, it would be interesting to use the same alphabetical materials in English to test native English speakers and further investigate whether language surface features affect semantic processing representations. In addition, an increase in sample size would further validate and support the findings of this study.

## 5. Conclusions

Overall, our results suggested that distributed networks of discrete cortical regions with distinct roles are active during semantic processing of logograms. The ventral occipitotemporal and inferior frontal regions display increased levels of encoding-related activity. The dorsal medial brain regions including superior frontal gyrus and occipitoparietal regions are associated with recognition-related activity.

## Figures and Tables

**Table 1:** Stereotactic Talairach coordinates, *t-*values, volume, and corresponding BAs for significantly activated regions (encoding versus baseline_1_ and recognition versus baseline_2_).

Comparison	Activated regions	BA	Coordinates	Peak *t-*values	Volume (mm^3^)
Encoding versus baseline_1_	R. inferior frontal gyrus	13/45/47	36, 24, 7	9.20	4,941
	R. middle frontal gyrus	46/9/8	56, 28, 26	6.42	7,857
	R. superior frontal gyrus	6	3, 11, 55	7.59	5,211
	L. middle frontal gyrus	10	−33, 56, 29	6.27	2,403
	R. claustrum	—	24, 26, 4	6.49	4,536
	Cingulate gyrus	24	3, 4, 25	4.79	2,916
	R. inferior parietal lobule	39/40	33, −62, 42	6.28	4,401
	R. superior parietal lobule	7	27, −53, 44	5.43	5,778
	R. fusiform gyrus	37/20	42, −62, −22	4.99	7,668
	R. inferior occipital gyrus	18/19	30, −93, −3	5.39	4,914

Recognition versus baseline_2_	R. superior frontal gyrus	6	6, 9, 58	8.54	7,020
	L. superior medial frontal gyrus	8/6/9/10/46	0, 20, 46	6.69	5,373
	L. inferior frontal gyrus	13/45/47	−33, 24, 7	6.68	3,078
	L. claustrum	—	−27, 21, 13	5.90	2,565
	R. superior parietal lobule	7	33, −50, 52	4.72	3,645
	R. inferior parietal lobule	40	39, −42, 33	4.30	2,592
	Cingulate gyrus	31	−18, −63, 14	5.28	4,671
	R. cuneus	18/19	6, −81, 12	6.57	6,561

L, left hemisphere; R, right hemisphere; BAs: Brodmann's areas.

Coordinates (*x*, *y*, *z*) are shown in Talairach space for the peak activation of each activated region. Peak *t-*values: peak intensity of voxel (*t*-score of peak voxel). Volume: numbers of voxel for the center-mass of peak activated region.

**Table 2:** Stereotactic Talairach coordinates, *t*-values, volume, and corresponding BAs for significantly activated regions (encoding versus recognition, recognition versus encoding).

Comparison	Activated regions	BA	Coordinates	Peak *t*-values	Volume (mm^3^)
Encoding versus recognition	L. inferior occipital gyrus	18/19	−30, −96, −3	9.22	7,965
	R. midinferior occipital gyrus	18/19	27, −90, −1	8.78	13,014
	L. fusiform gyrus	37/20/36	−39, −56, −12	5.16	16,848
	R. fusiform gyrus	37/20	42, −53, −10	6.78	21,762
	L. inferior frontal gyrus	47	−15, 31, −17	5.23	1,890
	R. superior medial frontal gyrus	11/25	21, 40, −20	5.11	4,671

Recognition versus encoding	L. precuneus	7	−3, −73, 45	8.50	12,285
	R. precuneus	7	6, −71, 39	8.06	15,417
	Cingulate gyrus	31/32	−18, −60, 17	7.57	12,285
	L. medial frontal gyrus	10	−6, 61, 5	7.26	10,827
	R. superior medial frontal gyrus	10	30, 56, 22	7.03	6,615
	L. middle temporal lobe	21	−45, −4, −12	4.95	8,559
	R. superior temporal gyrus	22	59, 12, −1	6.40	3,078
	L. inferior occipital gyrus	18/19	−18, −95, 27	5.69	8,181

L, left hemisphere; R, right hemisphere; BAs: Brodmann's areas.

Coordinates (*x*, *y*, *z*) are shown in Talairach space for the peak activation of each activated region. Peak *t*-values: peak intensity of voxel (*t*-score of peak voxel). Volume: numbers of voxel for the center-mass of peak activated region.
